# Descending necrotizing mediastinitis with esophageal perforation and tracheal ulcer: a case report and literature review

**DOI:** 10.1186/s44215-024-00135-9

**Published:** 2024-02-21

**Authors:** Yu Norimatsu, Naoki Enomoto, Daiki Kato, Shusuke Yagi, Kyoko Nohara, Kazuhiko Yamada, Norihiro Kokudo

**Affiliations:** Department of Surgery, National Center for Global Health and Medicine, 1-21-1 Toyama, Shinjuku-ku, Tokyo, 162-8655 Japan

**Keywords:** Descending necrotizing mediastinitis, Esophageal perforation, Esophagectomy

## Abstract

**Background:**

Descending necrotizing mediastinitis (DNM) is a rare and life-threatening condition, with high morbidity and mortality. Consequently, appropriate and prompt diagnosis and treatment are necessary. Herein, we report a case of esophageal perforation and bronchial ulcer secondary to DNM, for which thoracoscopic esophagectomy was performed after the failure of conservative treatment.

**Case presentation:**

A 63-year-old man was diagnosed with mediastinitis affecting the posterior mediastinum after presenting with a sore throat, back pain, and dyspnea. He also had type 2 diabetes mellitus and renal failure. The patient developed septic shock the following day, and intensive treatment was initiated. The condition of the patient gradually improved; however, the laboratory data revealed that levels of C-reactive protein remained high. On day 22, the esophageal perforation was demonstrated on esophagogastroduodenoscopy. Bronchoscopy was remarkable for the ulcer on the membranous tracheal wall, though he did not go into respiratory failure. The emergency thoracoscopic esophagectomy was performed on day 27. The reconstruction surgery was performed on day 100.

**Conclusions:**

Despite the fact that the general condition is stable with conservative treatment, DNM can develop esophageal perforation. Thus, it is essential to determine the appropriate timing of surgical intervention if levels of inflammation markers continue to be high.

## Background

Acute mediastinitis and esophageal perforation are medical emergencies with a high rate of morbidity and mortality. Descending necrotizing mediastinitis (DNM) is a rare and life-threatening form of mediastinitis which is a result from an oropharyngeal or cervical infection. The awareness of DNM among general surgeons is relatively low, despite the fact that the diagnosis of this condition relies on high suspicion and vigilance. In addition, detailed guidelines for the treatment of DNM have not yet been published [1–3]. Herein, we presented a case of DNM with perforation of the esophagus and aimed to discuss the treatment strategy of DNM.

## Case presentation

**Fig. 1:**
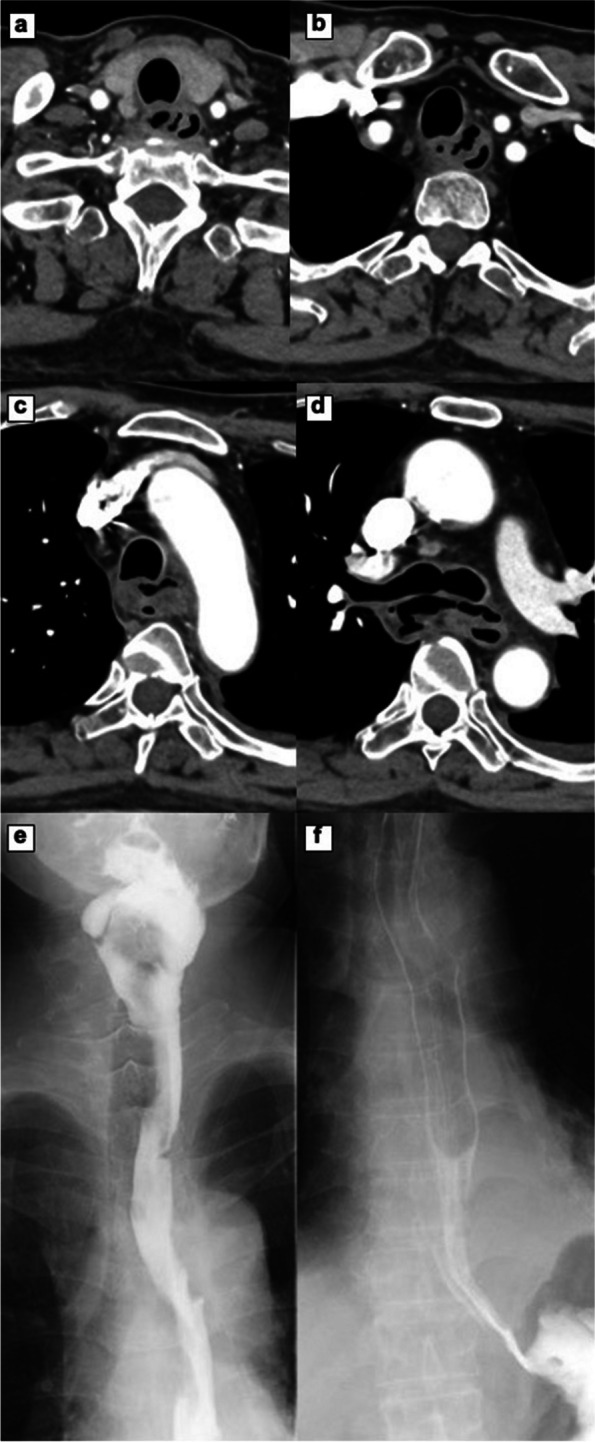
**a**–**d** CT revealed mediastinal emphysema and fluid accumulation around the cervical and thoracic esophagus. **e**, **f** Upper gastrointestinal radiography using gastrografin showed that there is no leak of the contrast from the esophagus. CT; computed tomography

**Fig. 2:**
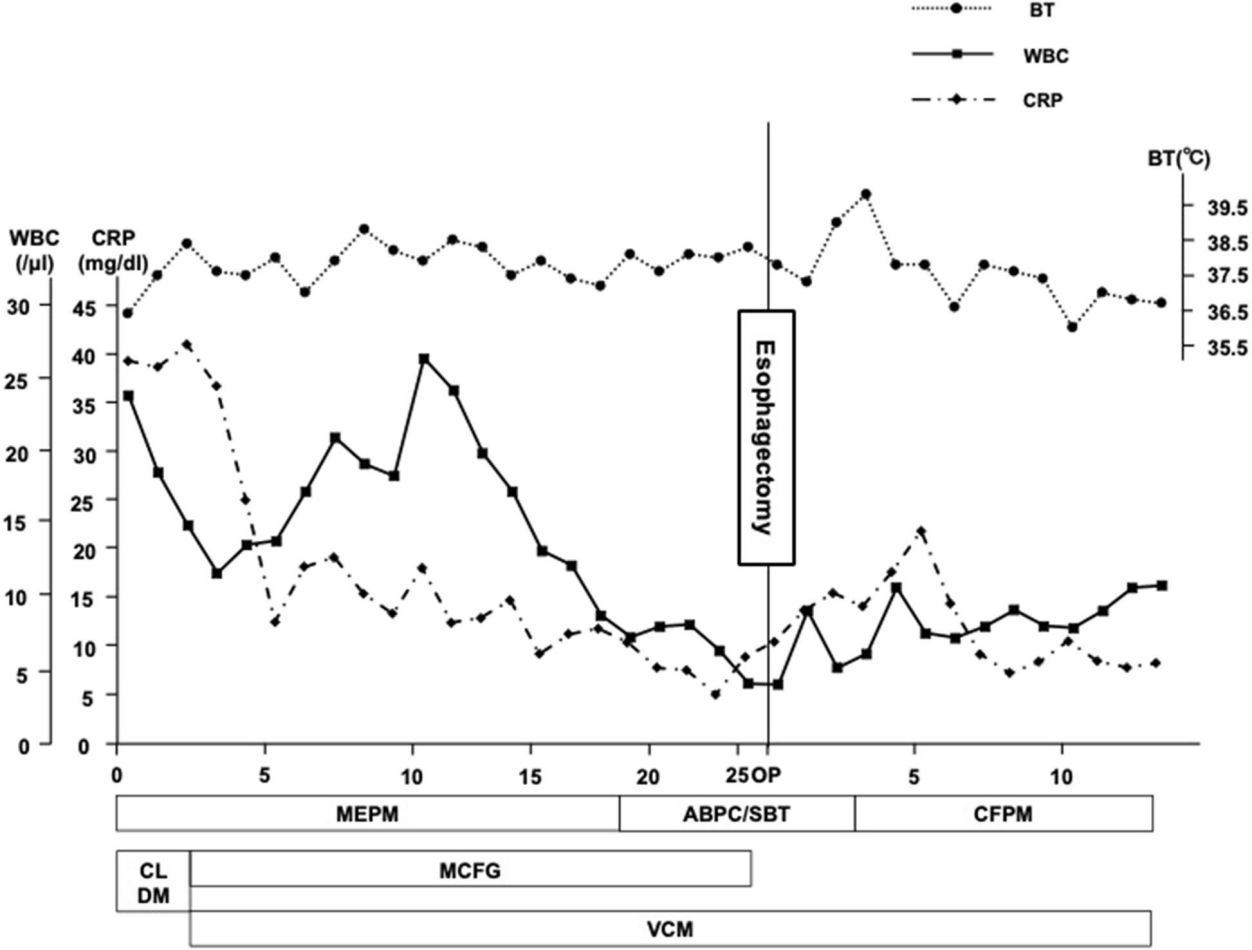
Trend of body temperature, levels of CRP and WBC, and antibiotics during the hospitalization. ABPC/SBT, ampicillin/sulbactam; CLDM, clindamycin; CRP, C-reactive protein; MCFG, micafungin; MEPM, meropenem; VCM, vancomycin; WBC, white blood cells

**Fig. 3:**
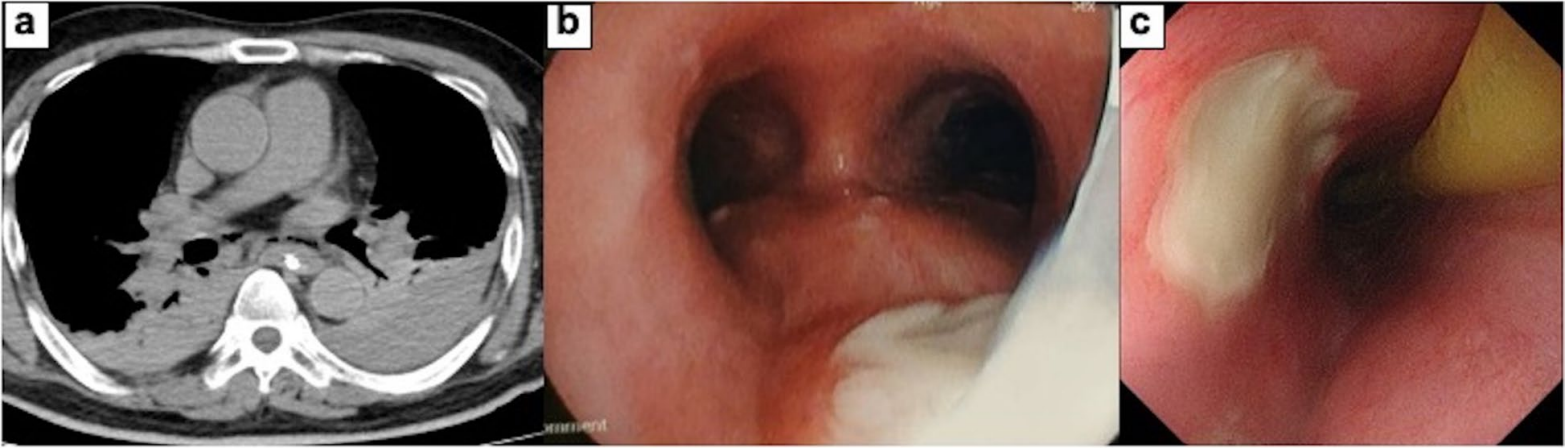
**a** CT revealed that mediastinal emphysema and pleural effusion in the left thoracic cavity had worsened on hospital day 2. **b** An ulcerative lesion above the tracheal bifurcation was observed on bronchoscopy. **c** EGD revealed ulcerative lesions with a size of 10 mm on the middle thoracic esophagus. EGD, esophagogastroduodenoscopy

A 63-year-old man visited our hospital with occasional back pain and dyspnea after suffering from sore throat and fatigue for a week. His medical history indicated that he had severe nephropathy due to type 2 diabetes mellitus, and he was undergoing dialysis three times a week. The blood pressure was slightly hypotensive (89/61 mmHg). The blood test revealed an elevated level of C-reactive protein (CRP) (39.3 mg/dl, normal rage 0.00–0.14), an increase in white blood cell count (23.8 × 10^3^ /μl, 3.30–8.60), renal dysfunction (blood urea nitrogen 25.6 mg/dl; 8.0–20.0, creatine 5.30 mg/dl; 0.65–1.07), and impaired glucose tolerance (hemoglobin A1c 7.5%; 4.6–6.2). Mediastinal emphysema and fluid accumulation in the cervical and upper thoracic mediastinum were seen on a computed tomography (CT) (Fig. 1a-d). However, upper gastrointestinal radiography revealed that there is no leak of the contrast from the esophagus (Fig. 1e, f). Initial treatments were started by inserting a nasogastric tube and administering broad-spectrum antibiotics (Fig. 2). Respiratory failure and hypotension developed on hospital day 1, and the patient was diagnosed with septic shock. The patient was intubated, and intensive treatment was started with a ventilator and vasopressor administration. Pleural effusion has increased in the left thoracic cavity as shown in the emergency CT (Fig. 3a). The chest tube was inserted into the left thoracic cavity, and the purulent pleural effusion was drained out. On day 4, the bronchoscopy was performed, and an ulcerative lesion on the tracheal carina was observed (Fig. 3b). On day 6, esophagogastroduodenoscopy (EGD) showed ulcerative lesions on the middle thoracic esophagus that measured 10 mm in size but did not appear to be perforated (Fig. 3c). The condition of the patient gradually improved, and on day 11, the patient was weaned off the ventilator.

**Fig. 4:**
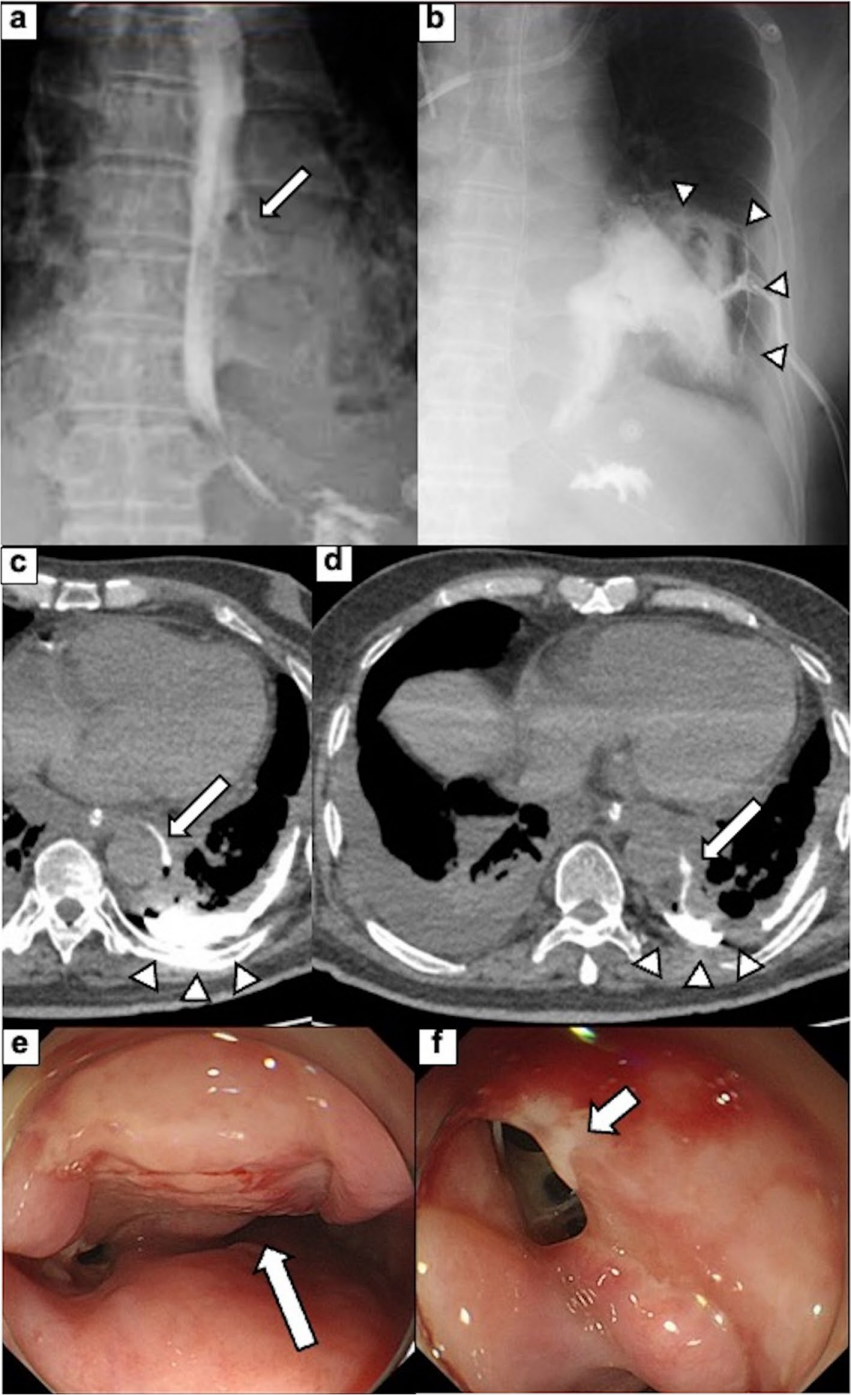
**a**, **b** Upper gastrointestinal radiography and (c, d) CT on hospital day 22. The findings revealed that there is leakage of the contrast from the esophagus to the left pleural cavity (a, c, and d, white arrow) and pleural effusion in bilateral thoracic cavities **b**, **c**, and **d**, white arrowhead). **e**, **f** EGD finding; the ulcer around the perforation (e, white arrow) and the perforation on the esophageal wall (f, white arrow)

**Fig. 5:**
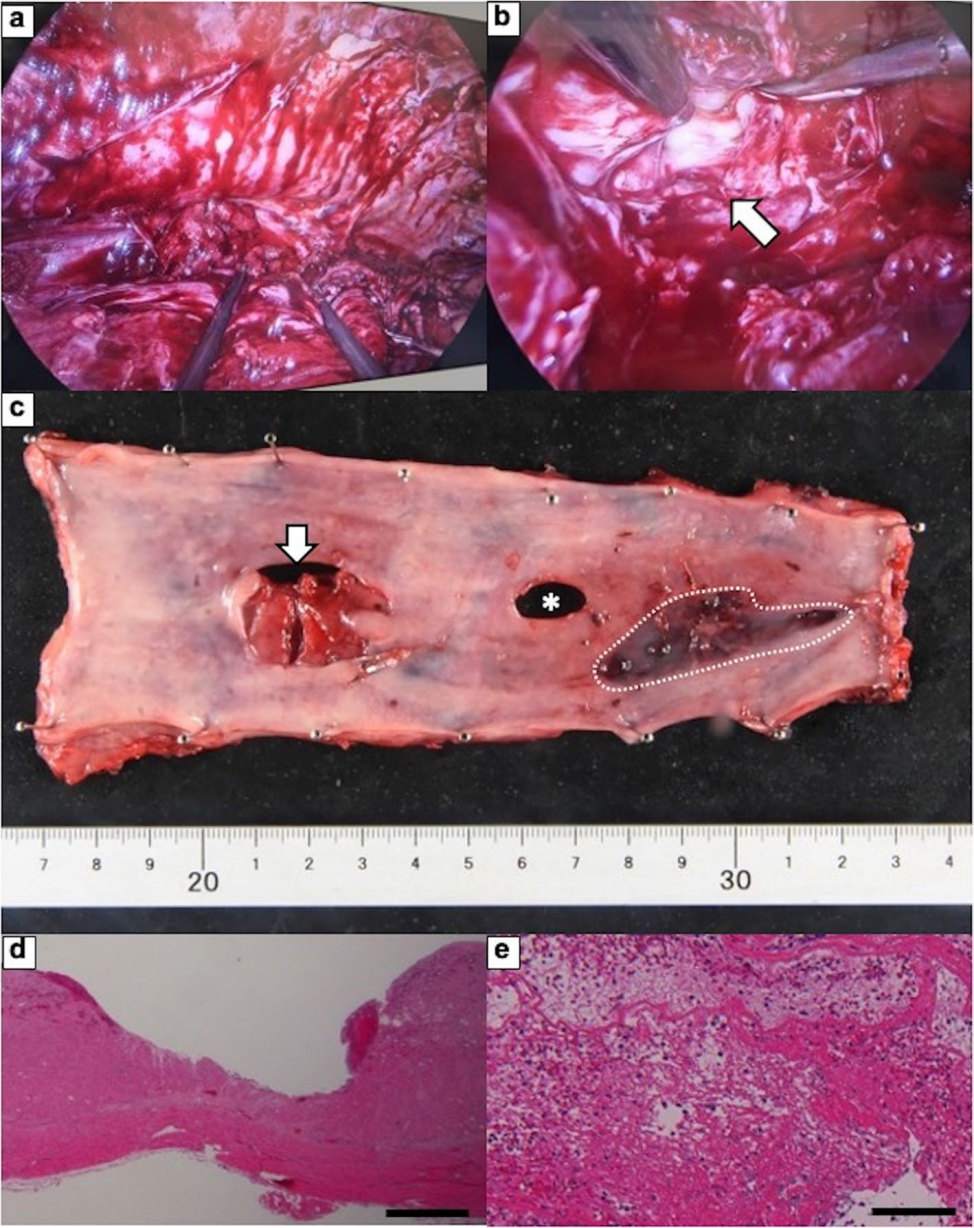
**a** Intraoperative photograph; severe adhesions in the thoracic cavity were carefully and patiently detached, although there were some bleeding observed. **b** Intraoperative photograph; a fistula connecting the esophageal perforation to the left thoracic cavity was exposed, and purulent fluid drained (arrow). **c** Surgical specimen. Esophageal ulcer with perforation with a size of 28 × 20 mm (arrow). The esophageal adventitia was detached from the esophagus to the trachea in the area enclosed by the dotted line. The hole (asterisk) was unintentionally created after the specimen was resected. **d**, **e** Pathological examination revealed inflammatory cell infiltration and granuloma formation. Hematoxylin and eosin stain. **d** × 20, and **e** × 40

Nevertheless, after ongoing treatment of antibiotics and drainage, the laboratory data showed high levels of CRP (Fig. 2). On radiography and the subsequent CT scan on day 22, the contrast media from the esophagus was leaked to the left chest cavity (Fig. 4a-d). Additionally, CT revealed a bilateral increase in the pleural effusion in thoracic cavities (Fig. 4c, d). The esophageal perforation was also demonstrated on EGD (Fig. 4e, f). Due to the limitations of conservative therapy, radical mediastinal drainage surgery was required. On day 27, emergency thoracoscopic esophagectomy was performed. Extensive adhesions throughout the thoracic cavity were observed (Fig. 5a, b). The adhesions were cautiously detached, and the esophagus was dissected from the surrounding tissue. There was also a fistula of the esophageal perforation into the left thoracic cavity. Purulent fluid from the fistula was observed. There was also severe adhesion between the esophagus and membranous trachea, where the tracheal ulcer was located. The adventitia was detached from the esophagus and covered the trachea without exposing it. The thoracic esophagus was resected, and the distal side of the cervical esophagus was diverted as an esophagostomy. Finally, a jejunostomy feeding tube was placed through the abdominal wall. The operative time was 313 minutes, and the estimated blood loss was 520 ml. On the surgical specimen, an esophageal perforation with a size of 28 × 20 mm was confirmed (Fig. 5c). Pathological examination showed esophageal ulcer and perforation with inflammatory cell infiltration and granuloma formation (Fig. 5d, e). On hospital day 100, the reconstruction surgery with gastric conduit via the antethoracic route was performed after the general condition had stabilized and the nutritional status had improved. Postoperatively, minor anastomotic leakage developed, but it was resolved with conservative treatment. Extended hospitalization, coupled with the consequences of two major surgeries in a short span, resulted in the patient progressing to an advanced stage of disuse syndrome. Subsequently, on day 224, the patient was transferred to a rehabilitation facility.

## Discussion

DNM often begins in the head or neck area, spreads quickly and aggressively along anatomic planes, and then settles into defined mediastinal compartments [4]. Although the etiology of DNM is still poorly understood, the most common cause of DNM is odontogenic infection [5, 6]. Risk factors for DNM include poor dentition, diabetes mellitus, malnutrition, advanced age, renal failure, and underlying malignancy [7, 8]. However, 13–30% of the patients do not have any comorbidities or predisposing factors [9]. Although this patient who had diabetes mellitus and renal failure complained of a week-long sore throat before developing the disease, otolaryngological and dental examinations conducted afterwards revealed no causal lesions in the oral cavity or pharynx. We diagnosed the patient with DNM because the mediastinal infection had spread rapidly and caudally.

When an aggressive infection spreads into the mediastinum following DNM, it can cause respiratory distress, sepsis, and death. Early recognition, diagnosis, and treatment are associated with good prognosis [10]. Nevertheless, DNM is not well recognized among general surgeons. Sugio and colleagues evaluated the clinical features and surgical outcomes of DNM, and they proposed a new classification system [11]. DNM was classified: infections confined to the area superior to the carina were categorized as type I, while infections spreading to the lower mediastinum (LM) were categorized as type II, with subcategories of type IIA for infections involving the anterior LM, type IIB for infections extended to both anterior and posterior LM, and type IIC for infections confined to the posterior LM. This classification was likely to contribute to clarify the relationship between the pattern of mediastinal infections and the surgical approach [12, 13]. The primary treatment currently involves drainage via transcervical or transthoracic procedures, although some authors believe that transcervical drainage alone is sufficient to control mediastinitis.

For type I DNM, any aggressive mediastinal drainage is not required, and a transcervical approach would be sufficient to control the infection. In contrast, for type IIA, subxiphoidal mediastinal drainage without sternotomy achieves adequate drainage, but for type IIB and IIC, complete mediastinal drainage through thoracotomy is often needed [11, 14, 15]. Our case was diagnosed as type IIC DNM because infections were limited to the posterior LM. It is possible that complications such as esophageal perforation would have been prevented if the patient had undergone transthoracic mediastinal drainage surgery at an earlier stage. However, the general condition of the patient was once significantly improved by conservative treatment, such as a thoracic tube placement. Given the risk posed by the comorbidities, we were unable to decide on the mediastinal drainage surgery when his health condition was stabilized.

Esophageal perforation caused by DNM has been extremely rare, and the exact etiology is still unknown [16]. Perforation in the esophagus and trachea due to DNM have been reported in eight cases since 1974, according to a literature review of English-language papers (Table 1) [17–24]. The reported cases include five cases of DNM-associated esophagus perforation. Notably, several DNM patients in previously reported cases exhibited comorbidities that influence peripheral vessels, including hypertension, angina pectoris, diabetes, and a history of dialysis. Additionally, three patients had perforation of the middle to lower thoracic esophagus. The cervical and upper thoracic esophagus receives blood from the inferior thyroid artery, whereas the middle thoracic esophagus are mostly supplied from the branches of the bronchial arteries [25]. The lower thoracic esophagus is fed by the small branches of the descending aorta. In addition to relatively low blood flow, the absence of supporting tissue around the esophagus may explain why esophageal perforations occur more frequently in the middle to lower thoracic esophagus than in other parts [26].

**Table 1:** Summary of reported cases of DNM with trachea or esophageal perforation

No.	Author	Year	Age/sex	Symptoms	Comorbidity	Origin of infection	Type^a^	Perforated organs	Treatments	Hospital day (m)	Outcome
1	Fukada J	1996	65/M	Chest pain, fever	None	Peritonsillar abscess	IIB	RMB	1st: Thoracotomic drainage	3	Survived
2	Kruyt PM	1996	41/M	Sore throat, fever	ND	Retropharyngeal abscess	IIC	UtE	1st: Conservative 2nd: Cervicomediastinal drainage	1.5	Survived
3	JR Roberts	1997	69/F	Dysphagia, palpable cervical emphysema	ND	ND	IIC	CeE	1st: Drainage by chest tube 2nd: Thoracoscopic drainage	1.5	Survived
4	Kato H	2000	77/M	Dyspnea, fever, neck swelling	ND	Dental caries	IIB	Trachea	1st: Cervicomediastinal and subxiphoid drainage 2nd: Thoracotomic drainage	8	Survived
5	Landers S	2007	67/M	Dysphagia, dyspnea	HT	Epiglottis	IIB	RMB	1st: Thoracoscopic drainage	7	Survived
6	Murakawa T	2010	34/M	Fever, neck swelling, sore throat	DM	Pharyngeal abscess	IIA	Trachea	1st: Cervicomediastinal datanage 2nd: Posterolateral thoracotomy with ECMO	5	Survived
7	Inaba Y	2010	78/M	Dysphagia, fever	Angina, HL	Peritonsillitis	IIC	MtE	1st: Conservative treatment	ND	Survived
8	Taha GE	2014	22/F	Dyspnea, fever, sore throat	DM	Acute bacterial pharyngitis	IIB	Trachea MtE	1st: Conservative 2nd: Cervicomediastinal drainage plus thoracotomy	4	Survived
9	Present case	2023	63/M	Back pain, dyspnea, sore throat	DM CRF	Unknown	IIC	LtE	1st: Consevative 2nd: Thoracoscopic esophagectomy	7	Survived

^a^Type of DNM classification system

*CeE* cervical esophagus: *CRF* chronic renal failure: *DM* diabetes mellitus: *ECMO* extracorporeal membrane oxygenation: *F* female: *HL* hyperlipemia: *HT* hypertension: *LtE* lower thoracic esophagus: *M* male: *MtE* middle thoracic esophagus: *ND* no data: *RMB* right main bronchus: *UtE* upper thoracic esophagus

Due to digestive contamination and subsequent damage to the surrounding organs, perforation of the esophagus can be life-threatening. Early diagnosis and timely therapeutic intervention are the keys to successful management, which includes conservative, and surgical therapies. The optimal treatment depends on the size of the rupture and the time to diagnosis. In recent years, minimally invasive surgery is increasingly performed not only in esophageal cancer but also in emergency cases such as esophageal perforation [27, 28]. Minimally invasive esophagectomy has been widely disseminated, as it brought reduced invasiveness and better postoperative quality of life than conventional open procedures [29]. Given the multiple comorbidities and high-risk nature of this case, we prioritized a minimally invasive strategy, opting for a thoracoscopic approach after ensuring optimal preoperative conditions. Our case demonstrated DNM with perforation of the lower thoracic esophagus and tracheal ulcer. It was assumed that the prolonged inflammation around the esophagus, anatomic paucity of blood flow, and the fragile nature of the tissue resulted in the perforation of the esophagus. The decision was made to perform an esophagectomy considering the large perforation size and the presumption that reoperation would be challenging in the event that esophagus-preserving surgery failed. Our strategy was to preserve the wall of the esophagus covering the tracheal ulcer, thereby mitigating the risk of tracheal perforation. In the event of an intraoperative tracheal perforation, our prepared approach encompassed the closure of the perforation site with suturing, complemented by reinforcement using either an intercostal muscle flap or a latissimus dorsi muscle flap. Intraoperative findings showed a strong adhesion between the trachea and the esophagus around the tracheal ulcer. However, perforation of the trachea was avoided by leaving the esophageal adventitia onto the tracheal side without forcibly detaching the adhesion. As reported in previous literature, intraoperative tracheal perforation is fatal [30, 31].

In conclusion, mediastinitis that spreads downward should be considered DNM. The comprehensive treatment strategy may reduce the mortality rate, which includes treatment with potent antibiotic and adequate drainage. Furthermore, DNM may lead to the perforation of the esophagus, especially in patients with severe comorbidities. Even if the patient appears to have a stable general condition, it is important that the appropriate timing for radical mediastinal drainage not be missed.

## Data Availability

The datasets during and/or analyzed during the current study are available from the corresponding author upon reasonable request.

## Abbreviations

DNM: descending necrotizing mediastinitis
CT: computed tomography
EGD: esophagogastroduodenoscopy
LM: lower mediastinum

## References

[1] Guan X, Liang X, Liang X, Wang F, Qian W, Zhang W. A new classification of descending necrotizing mediastinitis and surgical strategies. Ann translat med. 2021;9(4):356. 10.21037/atm-21-121 PMC7944333 33708983

[2] Novakov IP, Safev GP, Peicheva SE. Descending necrotizing mediastinitis of odontogenic origin-personal experience and literature review. Folia Med. 2010;52(3):13–20. 10.2478/v10153-010-0002-5 21053669

[3] Prado-Calleros HM, Jiménez-Fuentes E, Jiménez-Escobar I. Descending necrotizing mediastinitis: systematic review on its treatment in the last 6 years, 75 years after its description. Head Neck. 2016;38(S1):E2275–83. 26829352 10.1002/hed.24183

[4] Pearse HE Jr. Mediastinitis following cervical suppuration. Ann Surg. 1938;108(4):588. 17857255 10.1097/00000658-193810000-00009 PMC1387034

[5] Freeman RK, Vallieres E, Verrier ED, Karmy-Jones R, Wood DE. Descending necrotizing mediastinitis: an analysis of the effects of serial surgical debridement on patient mortality. J Thorac Cardiovasc Surg. 2000;119(2):260–7. 10649201 10.1016/S0022-5223(00)70181-4

[6] Hu CY, Lien KH, Chen SL, Chan KC. Risk factors of descending necrotizing mediastinitis in deep neck abscesses. Medicina. 2022;58(12):1758. 36556959 10.3390/medicina58121758 PMC9788205

[7] Taylor M, Patel H, Khwaja S, Rammohan K. Descending cervical mediastinitis: the multidisciplinary surgical approach. Eur Arch Otorrinolaringol. 2019;276(7):2075–9. 10.1007/s00405-019-05471-z 31093735

[8] Sumi Y. Descending necrotizing mediastinitis: 5 years of published data in J apan. Acute med surg. 2015;2(1):1–12. 29123684 10.1002/ams2.56 PMC5667189

[9] Kocher GJ, Hoksch B, Caversaccio M, Wiegand J, Schmid RA. Diffuse descending necrotizing mediastinitis: surgical therapy and outcome in a single-Centre series. Eur J Cardiothorac Surg. 2012;42(4):e66–72. 22761501 10.1093/ejcts/ezs385

[10] Wu P, Ye F, Zhang Z, et al. Descending necrotizing mediastinitis: analysis of 9 cases in our hospital. Ear Nose Throat J. 2021;100(5):350–3. 32627617 10.1177/0145561320933964

[11] Sugio K, Okamoto T, Maniwa Y, et al. Descending necrotizing mediastinitis and the proposal of a new classification. JTCVS open. 2021;8:633–47. 36004184 10.1016/j.xjon.2021.08.001 PMC9390273

[12] Bajpai S, Wei B. Commentary: descending necrotizing mediastinitis: reclassifying a rare disease. JTCVS open. 2021;8:650–1. 36004139 10.1016/j.xjon.2021.08.029 PMC9390264

[13] Cameron RB. Commentary: classifying descending necrotizing mediastinitis: what's the upshot? JTCVS open. 2021;8:648–9. 36004198 10.1016/j.xjon.2021.08.030 PMC9390388

[14] Misthos P, Katsaragakis S, Kakaris S, Theodorou D, Skottis I. Descending necrotizing anterior mediastinitis: analysis of survival and surgical treatment modalities. J Oral Maxillofac Surg. 2007;65(4):635–9. 17368356 10.1016/j.joms.2006.06.287

[15] Palma DM, Giuliano S, Cracchiolo AN, et al. Clinical features and outcome of patients with descending necrotizing mediastinitis: prospective analysis of 34 cases. Infection. 2016;44(1):77–84. 26335892 10.1007/s15010-015-0838-y

[16] Roccia F, Pecorari GC, Oliaro A, et al. Ten years of descending necrotizing mediastinitis: management of 23 cases. J Oral Maxillofac Surg. 2007;65(9):1716–24. 17719388 10.1016/j.joms.2006.10.060

[17] Fukada J, Inaoka M. A successful surgical case of descending necrotizing mediastinitis with fistula formation to the right main bronchus. Nihon kyobu geka gakkai zasshi. 1996;44(4):529–33. 8666874

[18] Kruyt PM, Boonstra A, Fockens P, Reeders JW, John J, van Lanschot B. Descending necrotizing mediastinitis causing pleuroesophageal fistula: successful treatment by combined transcervical and pleural drainage. Chest. 1996;109(5):1404–7. 8625701 10.1378/chest.109.5.1404

[19] Roberts JR, Smythe WR, Weber RW, Lanutti M, Rosengard BR, Kaiser LR. Thoracoscopic management of descending necrotizing mediastinitis. Chest. 1997;112(3):850–4. 9315828 10.1378/chest.112.3.850

[20] Kato H, Ohkubo N, Akazawa K, Iseki H, Haruna M. Spontaneous closure of a large tracheal fistula due to descending necrotizing mediastinitis. Ann Thorac Surg. 2000;69(4):1249–51. 10800830 10.1016/s0003-4975(99)01410-1

[21] Landers S, Beck A, Maurer J, Hurtgen M, Silomon M. Tracheobronchial necrosis. Following descending necrotizing mediastinitis. Anaesthesist. 2007;56(12):1237–41. 17901936 10.1007/s00101-007-1267-9

[22] Murakawa T, Yoshida Y, Fukami T, Nakajima J. Life-threatening tracheal perforation secondary to descending necrotizing mediastinitis. Interact Cardiovasc Thorac Surg. 2010;10(3):454–6. 19955171 10.1510/icvts.2009.225912

[23] Inaba Y, Tokano H, Ohtsu A, Kitamura K. A case of descending necrotizing mediastinitis penetrating to the esophagus. J rural med. 2010;5(2):190–3. 25648975 10.2185/jrm.5.190 PMC4309356

[24] Elsahy TG, Alotair HA, Alzeer AH, Al-Nassar SA. Descending necrotizing mediastinitis. Saudi med j. 2014;35(9):1123. 25228187 PMC4362155

[25] Geboes K, Geboes KP, Maleux G. Vascular anatomy of the gastrointestinal tract. Best pract res Clin gastroenterol. 2001;15(1):1–14. 11355897 10.1053/bega.2000.0152

[26] Augusto F, Fernandes V, Cremers M, et al. Acute necrotizing esophagitis: a large retrospective case series. Endoscopy. 2004;36(5):411–5. 15100949 10.1055/s-2004-814318

[27] Chirica M, Kelly MD, Siboni S, et al. Esophageal emergencies: WSES guidelines. World j emerg surg. 2019;14:1–15. 31164915 10.1186/s13017-019-0245-2 PMC6544956

[28] Eroğlu A, Aydın Y, Yılmaz Ö. Minimally invasive management of esophageal perforation. Turkish j thoracic cardiovas surg. 2018;26(3):496. 10.5606/tgkdc.dergisi.2018.15354 PMC7018280 32082789

[29] Biere SS, van Berge Henegouwen MI, Maas KW, et al. Minimally invasive versus open oesophagectomy for patients with oesophageal cancer: a multicentre, open-label, randomised controlled trial. Lancet. 2012;379(9829):1887–92. 22552194 10.1016/S0140-6736(12)60516-9

[30] Vikas G, Rajesh G, Shyam KST, Rana SS, Ashok KG, Sachin K, et al. Major airway injury during esophagectomy: experience at a tertiary care center. J Gastrointest Surg. 2009;13(3):438–41. 19002534 10.1007/s11605-008-0738-x

[31] Morita M, Saeki H, Okamoto T, Oki E, Yoshida S, Maehara Y. Tracheobronchial fistula during the perioperative period of Esophagectomy for esophageal Cancer. World J Surg. 2015;39(5):1119–26. 25588904 10.1007/s00268-015-2945-4

